# Assessing the impact of a combined analysis of four common low-risk genetic variants on autism risk

**DOI:** 10.1186/2040-2392-1-4

**Published:** 2010-02-22

**Authors:** Jerome Carayol, Gerard D Schellenberg, Frederic Tores, Jörg Hager, Andreas Ziegler, Geraldine Dawson

**Affiliations:** 1 IntegraGen SA, Evry, France; 2 Department of Pathology and Laboratory Medicine, University of Pennsylvania, Philadelphia, Pennsylvania, USA; 3 Department of Human Genetics, CEA-Genomics Institute, Evry, France; 4 Institute of Medical Biometry and Statistics, University of Luebeck, Luebeck, Germany; 5 Autism Speaks and the Department of Psychiatry, University of North Carolina at Chapel Hill, Chapel Hill, North Carolina, USA

## Abstract

**Background:**

Autism is a complex disorder characterized by deficits involving communication, social interaction, and repetitive and restrictive patterns of behavior. Twin studies have shown that autism is strongly heritable, suggesting a strong genetic component. In other disease states with a complex etiology, such as type 2 diabetes, cancer and cardiovascular disease, combined analysis of multiple genetic variants in a genetic score has helped to identify individuals at high risk of disease. Genetic scores are designed to test for association of genetic markers with disease.

**Method:**

The accumulation of multiple risk alleles markedly increases the risk of being affected, and compared with studying polymorphisms individually, it improves the identification of subgroups of individuals at greater risk. In the present study, we show that this approach can be applied to autism by specifically looking at a high-risk population of children who have siblings with autism. A two-sample study design and the generation of a genetic score using multiple independent genes were used to assess the risk of autism in a high-risk population.

**Results:**

In both samples, odds ratios (ORs) increased significantly as a function of the number of risk alleles, with a genetic score of 8 being associated with an OR of 5.54 (95% confidence interval [CI] 2.45 to 12.49). The sensitivities and specificities for each genetic score were similar in both analyses, and the resultant area under the receiver operating characteristic curves were identical (0.59).

**Conclusions:**

These results suggest that the accumulation of multiple risk alleles in a genetic score is a useful strategy for assessing the risk of autism in siblings of affected individuals, and may be better than studying single polymorphisms for identifying subgroups of individuals with significantly greater risk.

## Background

Autism is a heterogeneous disorder characterized by impairments in social interaction, deficits in verbal and nonverbal communication, restricted interests and repetitive behaviors [1]. Autism comprises the severe end of a group of autism spectrum disorders (ASD) [2]. The prevalence of autism is estimated at 0.2%, with males being more likely than females to have a diagnosis of autism (ratio of approximately 4:1) [3].

There is compelling evidence from twin and family studies indicating a strong genetic component in autism. The average risk of recurrence of autism in siblings is approximately 10% [4] in families with one affected sibling, which is much higher than the prevalence in the general population, but much lower than would be expected for a single-gene disease [2]. Indeed, cases of autism have only rarely been associated with mutations in single genes or with chromosomal duplications or other abnormalities [5-9]. Furthermore, the inheritance pattern in most cases of autism is complex and not compatible with simple Mendelian inheritance [10,11]. A series of common autism susceptibility genes has been identified in the literature, but taken individually, the risk associated with these genes remains modest. Although spontaneous mutations may help explain isolated cases of autism, the inheritance pattern of common variants may be at the root of autism in a large proportion of multiplex families.

Studies using simulated data have demonstrated that the joint analysis of such common low-risk variants has the potential to identify individuals at higher risk for disease, and could be useful for complex disease prediction [12-14]. In complex disease states such as type 2 diabetes [15-19], cancer [20,21] and cardiovascular disease [22-25], the accumulation of multiple risk alleles markedly increases the risk of being affected, and compared with studying single nucleotide polymorphisms (SNPs) independently, it is better at identifying subgroups of individuals with significantly greater risk. Previous studies have shown associations between several genetic variants and autism, but many of these studies could not be replicated in different populations.

For our study, we selected single nucleotide polymorphisms (SNPs) within four genes that have been previously shown to be associated with autism. Original publications and replication studies have shown that *SLC25A12 *[26-28] and *EN2 *[29,30] are associated with autism. *PITX1 *has been identified through a linkage study [31] using the physical identity by descent method [32]. The same method was used to identify *ATP2B2 *on chromosome 3 [33] in a region in which different autism linkage peaks and association with microsatellite markers have been reported [34-36]. We sought to demonstrate that joint analysis of common low-risk variants would have the potential to identify a subgroup of individuals at increased risk for developing autism. To assess the effect of combined analysis of multiple genetic variants in autism, we conducted a primary analysis on the first family sample using the four genes mentioned above. The aims of this analysis were to assess the strength of the association of the combination of these four genes with autism in a population of affected children, and to define a genetic scoring metric. We followed up with a second analysis to confirm our findings in an independent set of families, including a sibling diagnosed with autism.

## Methods

### Family samples

The study design involved two independent family samples. The first sample came from the Autism Genetic Resource Exchange (AGRE; http://www.agre.org) repository (AGRE sample), and consisted of 222 families with at least two affected offspring per family. In total, 527 affected siblings met the diagnostic criteria for 'narrow autism' according to the Autism Diagnostic Interview Revisited (ADI-R). The male to female ratio was 3.5:1, which reflected the gender distribution of autism in the general population. In this sample, all parents and affected children were genotyped at the four SNPs, whereas unaffected siblings were not genotyped.

The second (Seattle) sample was part of the University of Washington Family Study of Autism collection funded by the National Institute of Child Health and Human Development (NICHD), and consisted of 241 families with at least two offspring, at least one of whom was affected by autism. Additional criteria were used to categorize the 461 affected siblings with 'narrow autism,' including the ADI-R [37], the Autism Diagnostic Observation Schedule (ADOS-G) [38] scores, and a clinical diagnosis by an experienced clinician [39]. The male to female ratio was 3.7:1 and included 127 unaffected siblings as controls. The unaffected siblings were classified based on parental report in the Family History Interview [40] and the Broader Phenotype of Autism Symptom Scale [41].

In the Seattle sample, the IQ score as measured by the Wechsler scale in affected siblings was (mean ± SD) 76.3 ± 26.1, and age at IQ measurement was 9.4 ± 4.1. In unaffected siblings, the IQ score was 111 ± 16.3 with age at IQ measurement being 11.6 ± 6.4, which was comparable with the mean IQ score in parents (112.0 ± 14.0). All parents, affected siblings and unaffected siblings were genotyped at the four SNPs.

In the AGRE sample, single SNP analyses were conducted primarily with the goal of verifying that the preselected risk alleles corresponded with previous reports in the literature. The primary goal with the Seattle sample was to assess if the risk scores first generated in the AGRE sample could be replicated in the Seattle sample using 'true' sibling controls.

For both samples, exclusion criteria for children included a diagnosis of Rett syndrome and childhood disintegrative disorder, as defined by the *Diagnostic and Statistical Manual of Mental Disorders*, fourth edition (DSM-IV) criteria for other pervasive developmental disorders, presence of a known genetic condition, history of serious head injury or neurologic disease, or significant sensory or motor impairment [39]. Ethnicity was self-reported by parents as Caucasian, Asian, Hispanic or Latino, Black or African American, Native Hawaiian or other Pacific Islander, or of mixed ethnicities. Caucasians represented the major ethnicity, with more than two-thirds of families in each sample.

### Ethics approval

The study was approved by the respective institutional review boards (IRBs), and written informed consent was obtained from all authorized representatives. The AGRE sample had been approved by an IRB to ensure the protection of research participants. For the Seattle sample, all work with autism subjects was approved by the University of Washington Human Subjects Division Review Committees.

### SNP selection and genotyping

Four SNPs located in four separate genes from a pool of genetic polymorphisms that have been associated with autism were included in this multigene study: *rs2292813 *in *SLC25A12 *[26,27], *rs1861972 *in *EN2 *[29,30], *rs35678 *in *ATP2B2 *[33] and *rs6872664 *in *PITX1 *[31]. Genotyping was performed using TaqMan allele discrimination assays (Applied Biosystems, Foster City, CA, USA). Genotyping was performed in 384-well plates with 5 ng genomic DNA, 0.075 μL of 20 × SNP TaqMan Assay mix, 1.5 μL of TaqMan Universal PCR Master Mix and 1.425 μL of dH_2_O in each well. PCR was performed at 95°C for 10 minutes, followed by 50 cycles at 92°C for 15 seconds and 60°C for 90 seconds (9700 Gene Amp PCR System; Applied Biosystems). Plates were then subjected to end-point reading (7900 Real-Time PCR System; Applied Biosystems). Allele calls were assigned automatically, and a visual inspection of genotype clusters was performed. Genotyping quality was assessed by signal intensity plots and missing genotype frequencies; any samples with poor clustering and missing fractions ≥ 5% per SNP were re-typed. The genotyping success rate was found to be 97.4%. Parents' genotypes were used to investigate Hardy-Weinberg equilibrium (HWE) and to check for Mendelian inconsistencies. Families with remaining inconsistencies were excluded.

### Statistical analyses

Allelic frequencies were estimated for each variant in parents in both population samples. All analyses excluded the index case, defined as the oldest affected child in the family. Odds ratios (ORs), confidence intervals (CIs) and *P*-values were first calculated for all four variants individually. The sibling recurrence risk ratio attributable to the four markers was estimated as described previously [42]. Genetic scores for combinations of *SLC25A12, ATP2B2, EN2 and PITX1 *SNPs were then obtained using the allele count model [15,16,20,24], which takes into account all possible combinations of risk alleles by adding the number of alleles carried by each individual under an additive assumption expected for *ATP2B2 *(see Additional file 1). Individuals with an *ATP2B2 *risk allele had two points added to their genetic score if they were a homozygous carrier of the risk allele and zero points in all other cases, because the association of *ATP2B2 *with autism follows a recessive transmission model [33]. A subgroup analysis was conducted for the Caucasian families only because of the small sample sizes for all other ethnicities. Another subgroup analysis was performed separately for males and females using the risk score as an independent variable. For both subgroup analyses, the 'narrow' phenotype definition was used, and the analyses were conducted using the logistic regression iterative estimating equation (IEE).

Logistic regression was used to examine the association between the genetic score and the risk of autism, calculating the OR and CI associated with each additional risk allele in the genetic score. Following the definition of the reference group for single SNP OR estimates, the reference group for the risk score was chosen based on previous data [18,19,43] as the lower risk score. The discriminative ability of the model was evaluated, estimating sensitivity and specificity. The sensitivity describes the proportion of affected individuals having a genetic score above a given γ threshold. The specificity describes the proportion of unaffected individuals not having a genetic score above a given γ threshold. The receiver operating characteristic (ROC) curve was created by plotting sensitivity against 1 minus specificity, and the area under the curve (AUC) was calculated using the C-statistic.

The AGRE sample did not include genotyped unaffected siblings; specificity was not directly derived from data from unaffected individuals. We combined the case-pseudocontrol approach proposed by Cordell and Clayton [44,45] with a formula adapted from DeLong *et al*. [46], giving the specificity (spec) for a specific genetic score threshold γ as a function of the OR (ORγ) and the sensitivity (sens_γ_) as given in the equation below:

The OR associated with the γ threshold was calculated by conditional logistic regression using cases and their matched pseudocontrols. For each child with autism, a pseudocontrol was constructed from parental untransmitted alleles to the child with autism. Case-pseudocontrol statistical tests (developed by D. Clayton, Cambridge Institute for Medical Research; available at: http://www-gene.cimr.cam.ac.uk/clayton/software) were implemented within R (statistical program language). A version that was robust to non-independent siblings was implemented using the DGCgenetics R library, allowing for multiple affected offspring from the same nuclear family to be used in this study. In the Seattle sample, the analyses were performed as a case-control study in unaffected and affected siblings of the proband, using a generalized estimating equation (GEE) with a 'logit' link and the independence working correlation matrix to adjust for familial correlation [47]. The case-pseudocontrol design provides estimates of genetic relative risk [44,45] rather than OR, as in the case-control design. However, under the rare disease assumption, both estimates are equivalent.

A supplementary analysis using a GEE to correct for sibling relationship was conducted in the Seattle sample to check for a possible association between the genetic score and the Wechsler IQ scoring. Analyses were conducted first on the whole case-control Seattle sample, adjusting for gender and autism status, and in affected and unaffected siblings adjusting for gender.

## Results

**Figure 1:**
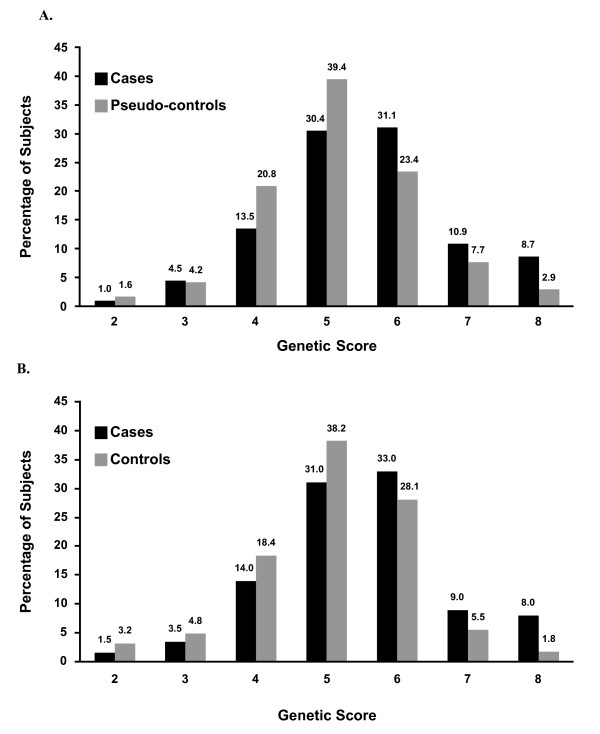
**Distribution of the genetic score in pseudocontrols, controls and cases**. Gray bars, distribution of **(a) **pseudocontrols or **(b) **controls; black bars, distribution of case subjects is indicated by black bars.

None of the SNPs exhibited a departure from HWE, and allele frequencies were similar between samples (Table 1). All risk alleles were very common, with frequencies ranging from 0.4 (*ATP2B2*) to 0.9 (*PITX*), similar to those estimated in the literature [26,27,29-31,33]. The distribution of case subjects in both samples was similar for each genetic score category (range 2 to 8), as was the distribution of pseudocontrol and control subjects. Control subjects were more often in the lower genetic score categories (≤ 4), and the case subjects were more often in the higher genetic score categories (> 6) (Figure 1).

**Table 1:** Risk allele frequency (defined as the allele associated with autism).

Gene	SNP	Risk allele	AGRE sample	Seattle sample
*PITX*	*rs6872664*	C	0.89	0.90
*ATP2B2*	*rs35678*	T	0.45	0.41
*SLC25A12*	*rs2292813*	C	0.90	0.90
*EN2*	*rs1861972*	A	0.75	0.72

AGRE, Autism Genetic Resource Exchange; SNP, single nucleotide polymorphism.

A primary analysis using the AGRE sample was performed to assess the association with autism of each individual SNP used in these analyses. The ORs for development of autism in siblings of affected children were calculated for each SNP using data from this sample. Nominally significant ORs were observed for the risk alleles for *EN2 *(*P *= 0.02), *PITX *(*P *= 0.01) and *ATP2B2 *(*P *= 0.01) (Table 2). Despite its reported association with autism [26-28], the OR for *SLC25A12 *did not reach significance (*P *= 0.38). An analysis was performed in which a genetic score was calculated (see Additional file 1), using the number of risk alleles carried by a subject, and testing for value in predicting the risk of autism in siblings of affected children (Table 3). A one-point increase in genetic score significantly increased the OR for autism in siblings of affected children (OR = 1.33; *P *< 0.0001). Individuals with a genetic score of 8 had an increased risk of being affected by autism (OR = 5.54; 95% CI 2.45 to 12.49) compared with individuals with two risk alleles (reference group). The same analysis was performed in the independent Seattle sample to confirm these findings. A similar distribution of genetic scores and ORs was observed in this analysis, with a one-point genetic score increase giving OR = 1.32 (95% CI 1.07 to 1.63) (Table 3). More than 50% of cases in both analyses had a genetic score of five or six risk alleles. In the AGRE sample, a genetic score of 5 or 6 gave ORs of 2.35 (95% CI 1.57 to 3.53) and 3.13 (95% CI 1.82 to 5.38), respectively, and in the Seattle sample, gave ORs of 2.30 (95% CI 1.23 to 4.31) and 3.04 (95% CI 1.32 to 7.01), respectively. Odds ratios for a one-point increase in the genetic scores in the Caucasian subgroup from the AGRE sample (OR = 1.39; 95% CI 1.17 to 1.66; see Additional file 2) and the Seattle sample (OR = 1.23; 95% CI 1.02 to 1.50; see Additional file 2) were similar to the ORs (1.33 and 1.32, respectively) estimated for the complete samples (Table 3). The ORs for gender subgroups were very similar in both samples (see Additional file 2).

**Table 2:** Results of genetic variants using the AGRE sample.

Gene	SNP	OR (95% CI)	*P*
*PITX*	*rs6872664*	1.58 (1.09 to 2.29)	0.01
*ATP2B2*	*rs35678*	1.74 (1.13 to 2.68)	0.01
*SLC25A12*	*rs2292813*	1.16 (0.83 to 1.64)	0.38
*EN2*	*rs1861972*	1.38 (1.06 to 1.79)	0.02

AGRE, Autism Genetic Resource Exchange; CI, confidence interval; OR, odd ratio; SNP, single nucleotide polymorphism.

**Table 3:** Results associated with genetic score.

Genetic score	OR (95% CI)	
	
	AGRE sample	Seattle sample
2^a^	1.00	1.00
3	1.33 (1.16 to 1.52)	1.32 (1.07 to 1.63)
4	1.77 (1.35 to 2.32)	1.74 (1.15 to 2.65)
5	2.35 (1.57 to 3.53)	2.30 (1.23 to 4.31)
6	3.13 (1.82 to 5.38)	3.04 (1.32 to 7.01)
7	4.16 (2.11 to 8.20)	4.01 (1.41 to 11.41)
8	5.54 (2.45 to 12.49)	5.30 (1.51 to 18.57)

AGRE, Autism Genetic Resource Exchange.

^a^Reference group.

**Figure 2:**
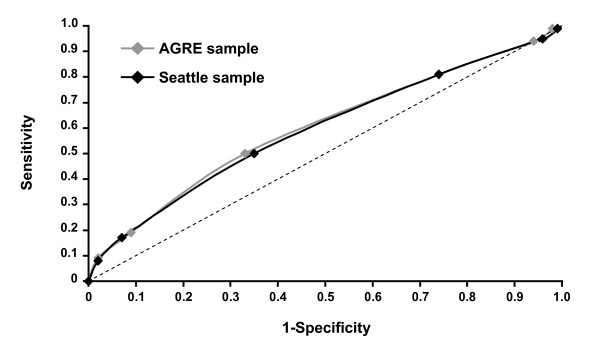
**Area under the receiver operating characteristic (ROC) curve (AUC) for the AGRE and Seattle samples**. ROC curve for the genetic score in the Autism Genetic Resource Exchange (AGRE) sample (gray line; AUC = 0.59, 95% CI 0.55 to 0.64) and the Seattle sample (black line; AUC = 0.59, 95% CI 0.53 to 0.65). Dashed line is reference value (AUC = 0.5).

The sensitivities and specificities for each genetic score were similar in the two samples, with overlapping CIs (Table 4). The resultant ROC curves for the genetic scores estimated in the two analyses (Figure 2) gave an AUC of 0.59 for both the AGRE (95% CI 0.55 to 0.64) and Seattle (95% CI 0.53 to 0.65) samples.

**Table 4:** Genetic score sensitivities and specificities predicting autism in siblings of affected children.

Genetic score	AGRE sample		Seattle sample	
	
	Sensitivity (95% CI)	Specificity (95% CI)	Sensitivity (95% CI)	Specificity (95% CI)
2*	1.00	0.00	1.00	0.00
3	0.99 (0.98 to 1.00)	0.02 (0.00 to 0.03)	0.99 (0.97 to 1.00)	0.01 (0.00 to 0.03)
4	0.94 (0.92 to 0.97)	0.06 (0.03 to 0.09)	0.95 (0.92 to 0.98)	0.04 (0.00 to 0.07)
5	0.81 (0.76 to 0.86)	0.26 (0.21 to 0.31)	0.81 (0.75 to 0.87)	0.26 (0.18 to 0.35)
6	0.50 (0.44 to 0.56)	0.67 (0.61 to 0.73)	0.50 (0.43 to 0.57)	0.65 (0.55 to 0.74)
7	0.19 (0.14 to 0.25)	0.91 (0.87 to 0.96)	0.17 (0.12 to 0.22)	0.93 (0.87 to 0.98)
8	0.09 (0.05 to 0.12)	0.98 (0.96 to 1.00)	0.08 (0.04 to 0.12)	0.98 (0.96 to 1.00)

AGRE, Autism Genetic Resource Exchange; CI, confidence interval.

^a^Reference group.

In the Seattle sample, for which true controls were available, 17% of individuals with autism carried seven risk alleles (genetic score ≥ 7) compared with only 8% of children without autism (Figure 1). Low sensitivity of 0.17 (95% CI 0.12 to 0.22) was associated with a high specificity of 0.93 (95% CI 0.87 to 0.98), which translated into a 2.33-fold increased risk for autism (Table 4). In the Seattle sample, 8% of cases had a maximum genetic score of 8, compared with only 2% of unaffected siblings of individuals with autism. The specificities for an extreme genetic score of 8 reached 0.98 (95% CI 0.96 to 1.00), but this was due to reduction in sensitivity (0.08; 95% CI 0.04 to 0.12), leading to a 4.44 increased risk of autism.

Based on these analyses and the distribution of the frequency of genetic score, we defined a low-score group of ≤ four risk alleles, which included approximately 23% of siblings, regardless of phenotype, in both samples. An intermediate-score group was defined as five or six risk alleles covering 62% and 65% of siblings and a high-score group of ≥ seven risk alleles covered 15% and 12% of siblings in the AGRE and Seattle samples, respectively. Using the low-score group as the reference group, the ORs for autism in the intermediate-risk group were 1.61 (95% CI 1.23 to 2.12) and 1.78 (95% CI 1.12 to 2.83) in the AGRE and the Seattle samples, respectively. Similarly, ORs for the high-risk group were 2.59 (95% CI 1.50 to 4.47) in the AGRE sample and 3.15 (95% CI 1.25 to 7.99) in the Seattle sample (Table 5).

**Table 5:** Results for the three genetic score groups.

Risk group (genetic score)	AGRE sample		Seattle sample	
	
	Case/pseudo- control frequencies	OR (95% CI)	Case/control frequencies	OR (95% CI)
Low (≤ 4)*	0.19/0.27	1.00	0.19/0.26	1.00
Intermediate (5 or 6)	0.61/0.63	1.61 (1.23 to 2.12)	0.64/0.66	1.78 (1.12 to 2.83)
High (≥ 7)	0.20/0.11	2.59 (1.50 to 4.47)	0.17/0.08	3.15 (1.25 to 7.99)

*Reference group.

We did not observe any association between the genetic score and IQ score in the whole case-control Seattle sample (*P *= 0.44), when adjusted for autism status and gender. This was also the case in affected (*P *= 0.63) and unaffected (*P *= 0.68) siblings adjusted for gender.

## Discussion

Complex pathologies such as autism may involve multiple common low-risk variants that confer relatively small predisposing effects. In the present study, we hypothesized that the combined analysis of such low-risk variants would more accurately identify a group of individuals at higher risk of developing the disease than an analysis of independent variants. To our knowledge, this is the first study report of its kind in autism.

Although many variants have been described in the literature as being associated with autism, few associations have been confirmed, possibly due to differences in methods and sample sets used, and to the weak effect of the individual variants. Previous studies have shown that variants in *SLC25A12 *[26-28], *EN2 *[29,30], *ATP2B2 *[33] and *PITX1 *[31], have an association with autism. A few studies have physiologically linked these genes to autism as well.

Expression of *SLC25A12 *may be involved in the pathophysiology of autism [48] because postmortem samples of brain tissue from patients with autism have been found to have stronger expression of *SLC25A12 *than normal brain samples. The *EN2 *gene is specifically involved in patterning the region that gives rise to the cerebellum [49]. *EN2 *knockout mice exhibit neuroanatomical and behavioral abnormalities that partly resemble those in patients with autism [50]. *PITX1 *is a key regulator of hormonal genes in the pituitary-hypothalamic axis. Its putative involvement in autism is supported by evidence documenting abnormal levels of downstream hormones, such as adrenocorticotropic hormone (ACTH), beta-endorphin and cortisol in individuals with autism [51-53]. Deregulation of pro-opiomelanocortin and high levels of beta-endorphin in the morning, for example, have been shown to be involved in certain maladaptive behaviors, such as self-injurious behaviors, which are often seen in individuals with autism [54]. The ACTH-cortisol system, which also plays an important role in stress-related responses, is impaired in individuals with autism in whom lower cortisol levels and higher ACTH levels have been reported [44,52]. Finally, the *ATP2B2 *gene codes for a major calcium pump expressed at particularly high levels in Purkinje neurons. Purkinje cells are involved in motor coordination, working memory and learning. Even though the exact correlation between Purkinje neurons and autism remains unclear, selective loss of Purkinje cells and cerebellar atrophies are the most consistently found neurological abnormalities in people diagnosed with autism [55].

Odds ratios ranging from 1.16 to 1.74 were observed in this study, and *P *values supported a nominal association for individual variants of *EN2*, *ATP2B2 *and *PITX1*, but not *SLC25A12*. However, because of its reported genetic association with autism [26,27,29] and the possible relationship with autism of its overexpression [48], it was included in the combined analysis. In the first stage of the combined assessment, a primary analysis was performed to evaluate the effect of the combination of variants as determined by the genetic score. In the second stage, a case-control analysis was performed to confirm the findings of the AGRE sample analysis. The distribution of genetic scores and ORs in this analysis was similar to that observed in the AGRE sample analysis, with an OR of 1.32. The ORs for a one-point increase in genetic score for the Caucasian subgroup in both the AGRE and Seattle sample analyses were also similar to those of the overall respective sample sets. We estimated that the OR for siblings who carry > seven risk alleles (approximately 12% of siblings), regardless of phenotype, was 3.15, compared with siblings who carry < four risk alleles (approximately 23% of siblings) in the Seattle sample, which used true sibling controls. A subgroup analysis did not yield any significant differences based on gender. Because of sample size, only the Caucasian subgroup could be analyzed. The ORs for a one-point increase in genetic score for the Caucasian subgroup in both the AGRE and Seattle sample analyses were also similar to those of the overall respective sample sets. We did not observe any association between IQ and genetic score, thus, we believe that the observed association is not an effect of the different IQ distributions between cases and controls.

The sensitivity and specificity for each genetic score was similar in both analyses. The resultant ROC curves for the genetic score estimated in these analyses produced an AUC of 0.59, significantly different (*P *< 0.01) from a random prediction of the score (AUC = 0.50). These results may be considered a first step toward the goal of using common genetic variants at multiple loci to develop a multigene approach to predicting the risk of autism. Indeed, these data are in line with other multigene risk assessment models in other complex diseases, such as type 2 diabetes, with reported AUCs of approximately 0.6 [15,16,19,56] using 3 to 18 common genetic variants. Results from these studies provide evidence that accumulation of multiple risk alleles markedly increases the risk of diabetes. However, compared with known nongenetic risk factors, such as body mass index, age and family history of the disease, combination of risk alleles provides only a relatively small increase in risk prediction [19,56,57]. In the case of autism, no early predictors exist to compare with genetic score. Nevertheless, there is significant interest in the early identification of infants at higher risk for autism because studies have shown that early intervention leads to significantly improved long-term outcome for the whole family [58,59].

The AUC is not a clinically relevant way of summarizing predictive performances. Furthermore, the AUC is a poor metric for evaluating markers for disease diagnoses, screening or prognosis [60]. Using a threshold of six risk alleles allows identification of 18% of cases (19% and 17% in the AGRE and Seattle samples, respectively) while reducing the likelihood of false-positive results (around 8%). Importantly, these sensitivity and specificity values translate into a > 2-fold increased risk for autism. In such a disorder, defined entirely on the basis of child behavior, this can be considered an important first step toward improved risk prediction.

Several studies support a strong genetic contribution to the etiology of autism, including twin studies that estimated a heritability of > 90% [61,62] and a family study that indicated a sibling recurrence risk ratio of 22 [63], which indicate that there is much potential for genetic prediction in autism. In the current study, the four SNPs used in the score are associated with a sibling recurrence risk ratio of 1.8. These results demonstrate that up to 10% of the variation in risk could be explained by these four genes.

Several strategies exist to identify relevant variants to be combined in a genetic score. These range from conservative approaches using only well-established factors [16,56,64,65] to more liberal approaches, which include polymorphisms from candidate genes or from regions that have already been associated with the disease or that display functional relevance [20,22,66]. We selected a subset of four variants from four candidate genes already associated with autism. An additional strategy would have been to include genes identified in a genome-wide association study [43]. The approach described here could be applied to other established variants and be part of future studies. Only one recent paper reported genome-wide scan results, which identified a common genetic variant reaching genome-wide significance located on 5p14.1 in the intergenic region between the *CDH10 *and *CDH9 *genes [67]. The reported ORs for the associated variant were similar to those of the variants in this study. However, it would be interesting to add this variant into the risk score. In our study, we chose the risk allele count as the most commonly used method of constructing genetic scores from low-effect genetic variants [15,16,20,24]. We used a recessive model to calculate the effect of *ATP2B2*, thereby strengthening our genetic score. Weighted scores have been proposed to increase discriminative power, but models that introduce information from variants with similar, small or moderate effect size only slightly increased the AUC [19,55].

One limitation of our study was that we assumed an individual effect for each gene. Gene by gene and/or gene by environment interaction should be tested and could be introduced into the genetic score for a more accurate assessment of risk associated with a given genetic score. Another limitation was that the polymorphisms used are probably not causal variants but rather markers in linkage disequilibrium. Causal variants may have different allele distributions in siblings with and without autism, and could have a stronger effect on disease risk. This is not yet clearly established and needs to be clarified [68].

Surprisingly, for three of the four loci investigated in this study, the major allele was overtransmitted in cases versus controls. Although it is unclear whether this same effect may hold for other autism-related risk loci, the observation is interesting and is one that merits further consideration in the field.

## Conclusions

In conclusion, the results of this study suggest that the joint analysis of common-risk variants will be more effective in identifying subgroups of individuals at risk than using single polymorphisms for risk assessment. The developed score could be improved by adding additional genes and environmental factors in the future.

## Competing interests

JC and FT are currently salaried employees of IntegraGen SA and JH is a former salaried employee. JC, FT and JH have stock options and patent applications with IntegraGen. GDS declares that he has no competing interests.

## Authors' contributions

JC, FT, JH, AZ and GD conceived and designed the experiments. GDS and GD performed the experiments. JC and FT analyzed the AGRE sample data and AZ analyzed the Seattle sample data. JC, FT and GDS contributed reagents, materials and/or analysis tools, and JC, FT, JH, AZ and GD assisted with writing of the manuscript.

## Supplementary Material

Additional file 1 **Supplementary Table 1**. Allelic variation used in the calculation of genetic score under an additive model. Click here for file

Additional file 2 **Supplementary Table 2**. Odds ratios (ORs) and 95% confidence intervals (CIs) associated with the genetic score in subpopulations. Click here for file
