# Supplementary material for: Assessing the impact of a combined analysis of four common low-risk genetic variants on autism risk
Source: Mol Autism. 2010 Feb 22;1:4. doi: 10.1186/2040-2392-1-4 (PMC2907567; doi:10.1186/2040-2392-1-4)
Supplement: Additional file 1 — Supplementary Table 1. Allelic variation used in the calculation of genetic score under an additive model. [file 2040-2392-1-4-S1.PDF]

**Supplementary Table 1. Allelic variation used in the calculation of genetic score under an additive model.**

| Gene | Risk Allele | Genetic Score = 0 | Genetic Score = 1 | Genetic Score = 2 |
|---|---|---|---|---|
| *PITX1* | C | TT | TC | CC |
| *ATP2B2** | T | CC/TC | -- | TT |
| *SLC25A12* | C | TT | TC | CC |
| *EN2* | A | GG | GA | AA |

*****Recessive transmission model.
