# Supplementary material for: Assessing the impact of a combined analysis of four common low-risk genetic variants on autism risk
Source: Mol Autism. 2010 Feb 22;1:4. doi: 10.1186/2040-2392-1-4 (PMC2907567; doi:10.1186/2040-2392-1-4)
Supplement: Additional file 2 — Supplementary Table 2. Odds ratios (ORs) and 95% confidence intervals (CIs) associated with the genetic score in subpopulations. [file 2040-2392-1-4-S2.PDF]

Supplementary Table 2. Odds ratios (ORs) and 95% confidence intervals (CIs) associated with the genetic score in subpopulations.

	AGRE Sample		Seattle Sample	
**Subgroup**	**OR**	**95% CI**	**OR**	**95% CI**
Caucasian	1.39	1.17-1.66	1.23	1.02-1.50
Caucasian, male only	1.35	1.10-1.65	1.35	1.04-1.75
Male only	1.35	1.15-1.57	1.35	0.89-2.05
Female only	1.30	0.95-1.77	1.31	1.03-1.65
